# Supplementary material for: CHiCAGO: robust detection of DNA looping interactions in Capture Hi-C data
Source: Genome Biol. 2016 Jun 15;17:127. doi: 10.1186/s13059-016-0992-2 (PMC4908757; doi:10.1186/s13059-016-0992-2)
Supplement: Additional file 1: — The mathematical specification of the CHiCAGO algorithm. (PDF 304 kb) [file 13059_2016_992_MOESM1_ESM.pdf]

CHiCAGO: Robust Detection of DNA Looping Interactions in Capture Hi-C data

Additional file 1: The mathematical specification of the CHiCAGO algorithm

Jonathan Cairns*, Paula Freire-Pritchett*, Steven W. Wingett, Csilla Várnai, Andrew Dimond, Vincent Plagnol, Daniel Zerbino, Stefan Schoenfelder, Biola-Maria Javierre, Cameron Osborne, Peter Fraser and Mikhail Spivakov

* Joint lead authors

Contents

1 Introduction **2**

2 Definitions **2**

3 Model **3**

4 Estimation – Brownian component **4**

4.1 Estimating $f(d)$ 5

4.2 Estimating s_j 6

4.3 Estimating s_i 6

4.4 Estimating r 7

5 Estimation – Technical noise **8**

6 Calculating *p*-values **8**

7 Working with multiple replicates **9**

8 Multiple testing and p-value weighting **10**
8.1 Estimation of Θ 12

A Mean bin value **14**

1 Introduction

This document describes, in detail, the statistical framework that underpins the CHiCAGO algorithm.

2 Definitions

- The genome is partitioned into *restriction fragments*, according to our choice of restriction enzyme. We follow the Hi-C protocol, making pairs of fragments ligate together. Subsequently, the capture step enriches for fragments of interest.

  A *bait* is a fragment that is captured by this last step. An *other end* (*OE*) is a fragment ligated to a bait. Thus, any fragment can be an OE, baits included.

- i, j are indices that refer to restriction fragments. i and j index over OEs and baits, respectively. With I OEs and J baits, we have $i \in \{1, \ldots, I\}$ and $j \in \{1, \ldots, J\}$. Since an OE can be a bait, we have that each baited fragment has both an i and j index.*

- Let X_{ij} be the number of observed read pairs that span from OE i to bait j.

- Let a "pair" be some choice (i, j).

- *cis* and *trans* are abbrevations for *cis-chromosomal* (same-chromosome) and *trans-chromosomal* (traversing from one chromosome to another), respectively.

*Note: in the software package, the indices i and j are not used – rather, each fragment gets an ID according to its genomic location. The ID is referred to as a baitID or otherEndID depending on context. Thus, in a typical list of all baitIDs, the numbers would not be contiguous.

- Let d_{ij} be the genomic distance, in base pairs, between the midpoints of fragments i and j (thus, $d_{ij} \geq 0$). If (i, j) is a trans pair, then we assume that d_{ij} is infinite.

- Often we need to group things by genomic distance. Thus, we define genomic distance bins of width w:

$$B_0 = [0, w), B_1 = [w, 2w), \ldots, B_b = [bw, (b+1)w)$$

  Let d_b be the midpoint of bin b. Thus, we can rewrite the definition of B_b as

$$B_b = [d_b - w/2, d_b + w/2)$$

- The Negative Binomial (NB) distribution will be parametrized throughout this document in terms of the mean μ and the dispersion parameter r (also known as the size parameter). Thus, if $X \sim NB(\mu, r)$,

$$Var(X) = \mu + \frac{\mu^2}{r}$$

3 Model

The aim of CHiCAGO is to find *interaction events*: pairs of loci that are brought together by some protein complex, in a manner that occurs more often than by chance were that complex not there. Under the null hypothesis (*ie* in the absence of such a complex), we assume that a count X_{ij} is a sum of two elements:

$$X_{ij} = B_{ij} + T_{ij}$$

These two components are:

- a "Brownian collision" component with NB distribution, $B_{ij} \sim NB(\mu_{ij}, r)$. This count represents read pairs that arise from the random collisions due to Brownian motion of the chromosome. Thus, the mean μ_{ij} decays with distance. We assume that μ_{ij} is the product of a bait fragment-specific bias s_j, an OE fragment-specific bias s_i, and some "distance profile" f that depends on the distance between the fragments:

$$\mu_{ij} = s_i s_j f(d_{ij})$$

where

$$f(d) \to 0 \text{ as } d \to \infty$$

Additional constraints are required to make this model identifiable (for example, the alternative solutions $s'_i = \alpha s_i$, $s'_j = \frac{s_j}{\alpha}$ have the same associated μ_{ij}). Thus, we normalise our scaling factors to have geometric mean 1:

$$\sum_i \log(s_i) = 0 \tag{1}$$

$$\sum_j \log(s_j) = 0$$

As a result, $f(d_{ij})$ represents the frequency of local interactions that an "average" bait would exhibit.

- a technical noise component, $T_{ij} \sim Pois(\lambda_{ij})$. This corresponds to reads introduced by assay artefacts, such as sequencing errors, and thus T_{ij} counts read pairs that did not arise from contact events. It is assumed that T_{ij} does not depend on distance. However, we permit complex non-multiplicative errors (see Section 5).

  In practice, we find that T_{ij} is very small.

We estimate each of the parameters f, s_j, s_i, r and λ_{ij} in turn.

4 Estimation – Brownian component

For a pair where the bait and OE are close, the technical noise is negligible compared to the Brownian component – that is, $\mu_{ij} >> \lambda_{ij}$.

Thus, under the null model, we can assume that

$$X_{ij} \sim NB(\mu_{ij}, r)$$

where

$$\mu_{ij} = s_i s_j f(d_{ij})$$

Initially, we aim to find the quantities s_j and $f(d)$. This must be done in a way that is robust against true interactions in the data.

4.1 Estimating $f(d)$

Note that, when estimating $f(d)$, we always ignore bait-to-bait pairs. That is, when summing over OEs i, we exclude all OEs i that are also baited fragments.

For convenience, we define genomic distance bins B_b that represent relative distances away from a bait. For the HindIII restriction enzyme, we set the bin width $w = 20$kb (approximately 5 restriction fragments), with the maximum distance at 1.5Mb by default (as specified by the *maxLBrownEst* setting). For a given bait j, we first calculate the average count over all of the OEs whose distance away from the bait lies in a given bin b:

$$\bar{X}_{bj} = \frac{1}{n_{bj}} \sum_{i; d_{ij} \in B_b} X_{ij}$$

where n_{bj} is the number of OEs in bin b.

We have that

$$\mathbb{E}(\bar{X}_{bj}) \approx s_j f(d_b)$$

(see Appendix, Section A.)

Any observations with $\bar{X}_{bj} = 0$ are censored. This is to ensure that zeros do not cause numerical instabilities in the next step.

We estimate $f(d_b)$ as the geometric mean count over all bins at distance d_b – that is,

$$\hat{f}(d_b) = geo_j \bar{X}_{bj}$$

or equivalently,

$$\log \hat{f}(d_b) = \frac{1}{J} \sum_j \log(\bar{X}_{bj})$$

This method is similar to the size factor estimation procedure in DESeq (Anders and Huber, 2010). We confirmed the accuracy with a simulation study (data not shown).

To get from $\hat{f}(d_b)$ to full inference of the function $\hat{f}(d)$, we fit a cubic function on a log-log scale using standard maximum likelihood estimation. We extrapolate linearly beyond the given d_b values assuming continuity of $f(d)$ and $f'(d)$.

4.2 Estimating s_j

When estimating s_j, bait-to-bait pairs are ignored as for $f(d)$ estimation. From the previous Section, we have

$$\mathbb{E}(\bar{X}_{bj}) \approx s_j f(d_b)$$

Thus, using our estimate $\hat{f}(d_b)$ from the previous section, a natural choice of estimator for s_j is $\bar{X}_{bj}/\hat{f}(d_b)$, for each b. Under the null hypothesis, for any b, the expected value of this is approximately s_j. However, if bin b contains many true interactions then this expectation no longer holds.

To avoid the influence of true interactions and gain a robust estimator of the s_j, we take the median as follows:

$$\hat{s}_j = median_b \frac{\bar{X}_{bj}}{\hat{f}(d_b)}$$

Note that this procedure also follows the DESeq model – specifically, the library size estimation procedure.

4.3 Estimating s_i

Our s_i estimation procedure is broadly similar to the s_j estimation procedure, but differs in one important aspect.

We have that

$$\mathbb{E}(X_{ij}) = s_j s_i f(d_{ij})$$

Consider a specific OE, i. An obvious estimator for s_i is the "normalised" count, $Y_{ij} = \frac{X_{ij}}{\hat{s}_j \hat{f}(d_{ij})}$. We could then take the median across js (i.e. across baits). However, this strategy fails, because we only get information about s_i for a small number of nearby baits. (Most baits have $Y_{ij} = 0$ and are therefore not very informative.) Indeed, if some of these baits j significantly interact with OE i, then s_i is greatly overestimated.

To address this, we "pool" OEs together according to how "noisy" they are. However, rather than assuming that noise specifically regresses against some arbitrary choice of explanatory variables, we take a data-driven approach where we postulate that the "noisiness" of each OE is reflected in the number of trans read pairs it is involved in, most of which are noise. Thus, we assign each OE i to a group $g(i)$, according to how many "trans" non-zero counts it has, and whether or not it is also a bait fragment:

$$g(i) = g \Leftrightarrow \begin{cases} \left(\sum_{j;(i,j)\ trans} I(X_{ij} > 0) \right) \in R_g \\ I(i \text{ is a bait}) = c_g \end{cases} \tag{2}$$

where:

- $I(.)$ is an indicator function.
- the groups R_g are defined such that each is a contiguous range of numbers, and such that there are approximately 1000 OEs per group/100 OEs per bait-to-bait group (by default). The exact implementation is according to the `cut2()` function (*Hmisc* CRAN package).
- c_g is 1 for a bait-to-bait group g, or 0 for a bait-to-non-bait group g.

It is assumed that all OEs in group g have approximately the same s_i. Thus, $s_i = s_{g(i)}$.

For each group, we calculate a per-distance bin estimate of s_g:

$$\bar{Y}_{gb} = \frac{\sum_{i;g(i)=g} \sum_{j;d_{ij} \in B_d} Y_{ij}}{\sum_{i;g(i)=g} \sum_{j;d_{ij} \in B_d} 1}$$

As before, we take the median across these bins:

$$\hat{s}_g = median_b(\bar{Y}_{gb})$$

4.4 Estimating r

We now calculate the dispersion, $\hat{r}$. This is simple to obtain from standard NB regression techniques – we find the r that maximises the likelihood of the regression model:

$$X_{ij} \sim NB(\mu_{ij}, r)$$

Since some of the pairs (i, j) are true interactions, there is slightly more variance across X_{ij} than there would be under the null, therefore we expect

$\hat{r}$ to be a slight underestimate of r. However, the number of interactions is very small compared to the number of pairs (we typically call around $1-2\%$ of pairs with $d_{ij} < 1.5$Mb), and thus this effect should be negligible. In any event, underestimation of r cannot introduce any false positives.

5 Estimation – Technical noise

Technical noise is assumed distance-invariant:

$$T_{ij} \sim Pois(\lambda_{ij})$$

The parameters λ_{ij} are estimated using trans pairs, since there is no contribution from the Brownian component, and thus $X_{ij} \approx T_{ij}$.

Because we have little information on λ_{ij}, we again pool fragments together, using a similar rationale to the s_i estimation procedure. OEs get classes $g(i)$ as before (Equation 2). However, this time, each bait j also gets a class $h(j)$ – as in Equation 2, the class is based on the number of OEs the bait interacts with in trans.

We assume that λ_{ij} depends only on the classes of i and j –

$$\lambda_{ij} = \Lambda(g(i), h(j))$$

To estimate Λ, we obtain all trans pairs with the appropriate class membership:

$$\Phi_{g,h} = \{(i,j); (i,j)\ trans, g(i) = g, h(j) = h\}$$

and calculate

$$\Lambda(g,h) = \frac{\sum\limits_{(i,j)\in\Phi_{g,h}} X_{ij}}{|\Phi_{g,h}|}$$

6 Calculating p-values

Putting the previous sections together we have that, under the null, X_{ij} has Delaporte distribution:

$$X_{ij} \sim NB(\mu_{ij}, r) + Pois(\lambda_{ij})$$

We perform a simple one-sided location test:

$$H_0 : \mathbb{E}(X_{ij}) = \mu_{ij} + \lambda_{ij}$$
$$H_1 : \mathbb{E}(X_{ij}) > \mu_{ij} + \lambda_{ij}$$

This test is performed using the *Delaporte* package, obtaining p-values $p_{ij} = p(X_{ij} \geq x_{ij})$.[†] CHiCAGO reports these p-values on the natural logarithmic scale.

7 Working with multiple replicates

We now consider the situation where multiple biological replicates are analysed simultaneously. The replicates are indexed by $k = 1, \ldots, K$. Thus, X_{ijk} is the count for OE i, bait j, replicate k.

We obtain sample-specific scaling factors s_k, in a manner akin to that of DESeq (Anders and Huber, 2010) by looking at regions proximal to baits. The procedure is:

- Take a window around each bait (by default, 1.5Mb in either direction, as specified by the *maxLBrownEst* setting)
- Count number of reads, divide by number of OEs present, to get M_{jk}.
- Take geometric mean across samples. $G_j = geo_k(M_{jk})$
- $s_k = median_j(M_{jk}/G_j)$

A summarised count is calculated as a weighted average of the individual samples' counts:

$$X_{ij} = round\left(\frac{\sum_k s_k X_{ijk}}{\sum_k s_k}\right)$$

[†]In some rare situations, x_{ij} was too large compared to $E(X_{ij})$, and we encountered underflow issues. Here, we approximated X_{ij} by a Negative Binomial distribution, using the Method of Moments. In other words, we assume that, under the null,

$$X_{ij} \approx X'_{ij} \sim NB(\eta_{ij}, \rho_{ij})$$

where η_{ij} and ρ_{ij} are found by equating the mean and variance of $\mathbb{E}(X)$ and $\mathbb{E}(X')$.

This has parallels to pooling biological replicates (as has been common in ChIP-seq data, for example), but rather than simply taking the total number of reads, we use a more appropriate estimator of library size (akin to "effective library size" in RNA-seq) that is primarily driven by the Brownian component.

This summarised count is taken forward in the analysis.

We can also derive normalised counts $\tilde{X}_{ijk} = \frac{1}{s_k} X_{ijk}$, which can be useful for visualisation purposes.

8 Multiple testing and *p*-value weighting

We expect far more interactions to occur at short ranges than at long ranges. However, suppose that we call interactions by applying a threshold directly to p-values. Of the hypotheses we test, a large majority are long-range interactions. Thus, with more opportunities to return a p-value below the threshold by chance, our output is dominated by erroneous long-range calls. Another way to look at this is that, when ordering p-values, there are sufficiently many long-range interactions with lower p-values than true short-range interactions that we cannot call the short-range interactions without accepting the long-range false positives as well.

Standard multiple testing procedures fail to address this problem. For example, the Bonferroni and Benjamini-Hochberg methods both choose a stringent p-value threshold – as described above, this may discard the long-range false positives, but we also lose many short-range true positives.

A number of relevant approaches are described in Gui *et al.* (2012). For example, Sun *et al.* (2006) use a two-population approach, which we could apply by splitting our hypotheses in two using a distance threshold. However, this method is very sensitive to the choice of distance threshold. Moreover, it also assumes a sudden change of behaviour, which is not biologically plausible as there appears to be a more gradual change in behaviour. Thus, we chose the Genovese *et al.* (2006) approach, p-value weighting, which is a generalized version of Sun *et al.* (2006).

We also considered the use of an empirical Bayes treatment, where a prior probability is used to quantify the two behaviours. However, the Bayesian approach requires explicit assumptions of the read distribution under the alternative hypothesis, over and above requiring a larger mean. p-value weighting can be viewed as a simplified version of an empirical Bayesian treatment,

using a "weight" in place of a prior probability. This method circumvents the need to make an arbitrary choice of the prior distribution of read counts under the alternative hypothesis.

The aim of the p-value weighting strategy is to "upweight" the significance of proximal pairs and "downweight" distal/trans pairs. Using the notation in GENOVESE *et al.* (2006), we make prior "guesses" U_{ij}. We allow U_{ij} to depend on d_{ij}, assuming that short-range interactions are more likely than long-range interactions, with a smooth transition between the two. The U_{ij} are transformed into weights W_{ij} by dividing through by the mean value, $\bar{U}$, ensuring that the average W_{ij} value is 1. Finally, weighted p-values are obtained by dividing the p-values by their respective weights:

$$Q_{ij} = \frac{p_{ij}}{W_{ij}}$$

We now specify the U_{ij} model in our particular context. We use a bounded logistic regression model – thus, U_{ij} is assumed a function of both d_{ij} and a vector of parameters $\Theta = (\alpha, \beta, \gamma, \delta)$, according to

$$U_{ij} = \eta_{ij} U_{\max} + (1 - \eta_{ij}) U_{\min}$$

where

$$\begin{aligned} \eta_{ij} &= \text{expit}(\alpha + \beta \log(d_{ij})) \\ U_{\min} &= \text{expit}(\gamma) \\ U_{\max} &= \text{expit}(\delta) \end{aligned}$$

using the expit function

$$\text{expit}(x) = \frac{e^x}{1 + e^x}$$

A method for choosing Θ is discussed in the next subsection. To obtain the weights, we first need to calculate $\bar{U}$, the mean value of U_{ij}:

$$\begin{aligned} \bar{U} &= \frac{1}{m} \sum_i \sum_j U_{ij} \\ &= \bar{\eta} U_{\max} + (1 - \bar{\eta}) U_{\min} \end{aligned}$$

Hence, we calculate the weights W_{ij} as follows:

$$\begin{aligned} W_{ij} &= \frac{U_{ij}}{\bar{U}} \\ &= \frac{\eta_{ij} U_{\max} + (1-\eta_{ij}) U_{\min}}{\bar{\eta} U_{\max} + (1-\bar{\eta}) U_{\min}} \\ &= \frac{\eta_{ij} U_{\text{rel}} + (1-\eta_{ij})}{\bar{\eta} U_{\text{rel}} + (1-\bar{\eta})} \end{aligned}$$

where $U_{\text{rel}} = \frac{U_{\max}}{U_{\min}} = \frac{\text{expit}(\gamma)}{\text{expit}(\delta)}$. We now use W_{ij} to calculate weighted p-values:

$$Q_{ij} = \frac{p_{ij}}{W_{ij}}$$

CHiCAGO reports these values on the log-scale.

Genovese *et al.* (2006) obtain a false discovery rate, by applying the Benjamini-Hochberg procedure to their weighted p-values. Unfortunately, the requirements required for Benjamini-Hochberg are not satisfied: since our data are discrete (in particular, many counts are 0), we do not have uniform p-values under the null hypothesis. Thus, our preferred strategy is to set a threshold on the Q_{ij} values.

To aid interpretation, we also compute a score based on Q_{ij} as follows:

$$score_{ij} = \max(0, -\log Q_{ij} - \log W_{\max})$$

where $W_{\max}$ is the value that W_{ij} would take when $d_{ij} = 0$.

In other words, the score is non-negative, and a positive score occurs only when the evidence for an interaction exceeds that of a proximal pair with no reads.

For most users' analyses, the score will be the most appropriate quantity to threshold on.

8.1 Estimation of Θ

The above depends on the unknown parameter vector, Θ. We can estimate Θ from a candidate data set, provided that it has multiple biological replicates, as follows. We split the data into subsets that contain approximately equal numbers of baits. (By default, 5 subsets are used.) The reproducible interactions are defined as those where the stringent threshold of $\log(p) < -10$ is

passed in all biological replicates. Now, for each subset, we take a series of genomic distance bins (with the default breaks occurring at 0, 31.25k, 62.5k, 125k, 250k, 500k, 1M, 2M, 3M, 4M, ..., 16M), and we calculate the proportion of reproducible interactions out of the total number of possible interactions. The maximum likelihood estimates are calculated for each model parameter, using standard optimization methods (Nelder and Mead, 1964).

Final parameter estimates are obtained by taking the median across the estimates from each subset, in a component-wise manner. That is, if $\hat{\Theta}_i^s$ is the estimate for parameter i on subset s, then our final parameter estimate is:

$$\hat{\Theta}_i = median_s(\hat{\Theta}_i^s)$$

This is to ensure robustness against any large-scale interactions in the data.

At time of writing, users may choose from three different Θ estimates in the package, as follows:

- GM12878 data, over two replicates (Mifsud *et al.*, 2015).
- mESC data, over two replicates (Schoenfelder *et al.*, 2015).
- Human macrophage data, over seven replicates (Javierre *et al.*, in preparation).

The macrophage weights are used by default. If a user wishes to use CHiCAGO on cells whose interactomes are expected to differ greatly from the provided cell types, they can estimate a new Θ using *fitDistCurve.R* in *chicagoTools*.

References

Anders, S., and W. Huber, 2010 Differential expression analysis for sequence count data. Genome Biology **11**: R106.

Genovese, C. R., K. Roeder, and L. Wasserman, 2006 False discovery control with p-value weighting. Biometrika **93**: 509–524.

Gui, J., T. D. Tosteson, and M. Borsuk, 2012 Weighted multiple testing procedures for genomic studies. BioData mining **5**: 4.

Mifsud, B., F. Tavares-Cadete, A. N. Young, R. Sugar, S. Schoenfelder, *et al.*, 2015 Mapping long-range promoter contacts in human cells with high-resolution capture Hi-C. Nature genetics **47**: 598–606.

Nelder, J. a., and R. Mead, 1964 A simplex method for function minimization. The Computer Journal **7**: 308–313.

Schoenfelder, S., M. Furlan-Magaril, B. Mifsud, F. Tavares-Cadete, R. Sugar, *et al.*, 2015 The pluripotent regulatory circuitry connecting promoters to their long-range interacting elements. Genome research **25**: 582–597.

Sun, L., R. V. Craiu, A. D. Paterson, and S. B. Bull, 2006 Stratified false discovery control for large-scale hypothesis testing with application to genome-wide association studies. Genetic Epidemiology **30**: 519–530.

A Mean bin value

In this Section, we aim to show that

$$\mathbb{E}(\bar{X}_{bj}) \approx s_j f(d_b)$$

To do this, we use a mixed effects model. s_j is assumed a fixed effect, whereas s_i and d_{ij} are assumed to be independent random effects:

- $\log s_i$ has some random distribution with mean 0 and variance σ_s^2. We therefore have that

$$\begin{aligned}\mathbb{E}(s_i) &= \mathbb{E}(e^{\log s_i}) \\ &= \mathbb{E}\left(1 + \log s_i + \frac{(\log s_i)^2}{2} + \dots\right) \\ &\approx 1 + \frac{\mathbb{E}\left((\log s_i)^2\right)}{2} \\ &= 1 + \frac{\sigma_s^2}{2}\end{aligned}$$

- For $b > 1$, it is reasonable to assume that, conditional on $d_{ij} \in B_b$,

$$d_{ij} \sim U\left(d_b - \frac{w}{2}, d_b + \frac{w}{2}\right) \tag{3}$$

which means that $\mathbb{E}(d_{ij}) = d_b$ and $var(d_{ij}) = \frac{w^2}{12}$. Therefore,

$$f(d_{ij}) = f(d_b) + (d_{ij} - d_b)f'(d_b) + \frac{(d_{ij} - d_b)^2}{2} f''(d_b) + \dots$$
$$\mathbb{E}\left(f(d_{ij})\right) = f(d_b) + \mathbb{E}(d_{ij} - d_b)f'(d_b) + \mathbb{E}\left(\frac{(d_{ij} - d_b)^2}{2}\right) f''(d_b) + \dots \tag{4}$$

Using assumption 3 allows us to simplify this equation, since the symmetry of d_{ij}'s distribution means that terms featuring odd powers cancel out. We also assume that $f(d)$ is sufficiently smooth that the terms featuring fourth and higher derivatives are negligible.[‡] Thus, Equation 4 simplifies down to:

$$\begin{aligned} \mathbb{E}\left(f(d_{ij})\right) &\approx f(d_b) + \mathbb{E}\left(\frac{(d_{ij} - d_b)^2}{2}\right) f''(d_b) \\ &= f(d_b) + \frac{w^2}{24} f''(d_b) \end{aligned}$$

We represent this in the form:

$$\mathbb{E}\left(f(d_{ij})\right) \approx f(d_b)\left(1 + \epsilon_b\right)$$

where ϵ_b represents the proportion of error introduced by change in f, for bin b. So, for the case $b > 1$, we have that $\epsilon_b = \frac{w^2}{24}\frac{f''(d_b)}{f(d_b)}$.

- For $b = 1$, assumption 3 is violated when fragments adjacent to the bait are removed (*i.e. removeAdjacent = TRUE*), which is the recommended setting for non-binned data. In this case, d_{ij} is non-uniform, and thus $\mathbb{E}(d_{ij} - d_b) \neq 0$. As a result, Equation 4 cannot be simplified in the same way as before. However, we can calculate the expectations in Equation 4 using the empirical distribution of d_{ij}, obtained from the data, to get an estimate of ϵ_1.

Claim:

$$\mathbb{E}(\bar{X}_{bj}) \approx s_j f(d_b)\left(1 + \frac{\sigma_s^2}{2}\right)(1 + \epsilon_b)$$

[‡]More specifically, for $k = 4, 6, 8, \dots$, we require that $\frac{f^{(k)}(d_b)}{f^{(k-2)}(d_b)}\frac{w^2}{4k(k+1)}$ is small enough that the larger order terms in the Taylor expansion can be omitted. We verified this assumption numerically on the distance function estimate from the GM12878 data. (Mifsud *et al.*, 2015)

Proof:

$$
\begin{aligned}
\mathbb{E}(\bar{X}_{bj}) &= \frac{1}{n_{bj}} \sum_{i;d_{ij}\in B_b} \mathbb{E}(X_{ij}) \\
&= \frac{1}{n_{bj}} \sum_{i;d_{ij}\in B_b} \mathbb{E}(\mathbb{E}(X_{ij}|d_{ij}, \log s_i)) \\
&= \frac{1}{n_{bj}} \sum_{i;d_{ij}\in B_b} \mathbb{E}(s_i s_j f(d_{ij})) \\
&= \frac{s_j}{n_{bj}} \sum_{i;d_{ij}\in B_b} \mathbb{E}(s_i)\mathbb{E}(f(d_{ij})) \\
&\approx \frac{s_j}{n_{bj}} \sum_{i;d_{ij}\in B_b} \left(1 + \frac{\sigma_s^2}{2}\right) f(d_b)\,(1+\epsilon_b) \\
&= s_j f(d_b)\left(1 + \frac{\sigma_s^2}{2}\right)(1+\epsilon_b)
\end{aligned}
$$

We estimated the bias terms in the merged GM12878 dataset (Mifsud *et al.*, 2015), obtaining negligible biases:

$$\frac{\hat{\sigma}_s^2}{2} = 0.0019$$

and

$$\epsilon = \begin{pmatrix} -0.019 \\ 0.014 \\ 0.0053 \\ 0.0029 \\ 0.0018 \\ \vdots \\ 4.2 \times 10^{-6} \end{pmatrix}$$
