# Supplementary material for: CHiCAGO: robust detection of DNA looping interactions in Capture Hi-C data
Source: Genome Biol. 2016 Jun 15;17:127. doi: 10.1186/s13059-016-0992-2 (PMC4908757; doi:10.1186/s13059-016-0992-2)
Supplement: Additional file 2: — Figures S1 to S6 and Table S1. (PDF 803 kb) [file 13059_2016_992_MOESM2_ESM.pdf]

CHiCAGO: Robust Detection of DNA Looping Interactions in Capture Hi-C data

Additional file 2: Figures S1 through S6, and Table S1

Jonathan Cairns*, Paula Freire-Pritchett*, Steven W. Wingett, Csilla Várnai, Andrew Dimond, Vincent Plagnol, Daniel Zerbino, Stefan Schoenfelder, Biola-Maria Javierre, Cameron Osborne, Peter Fraser and Mikhail Spivakov

* Joint lead authors

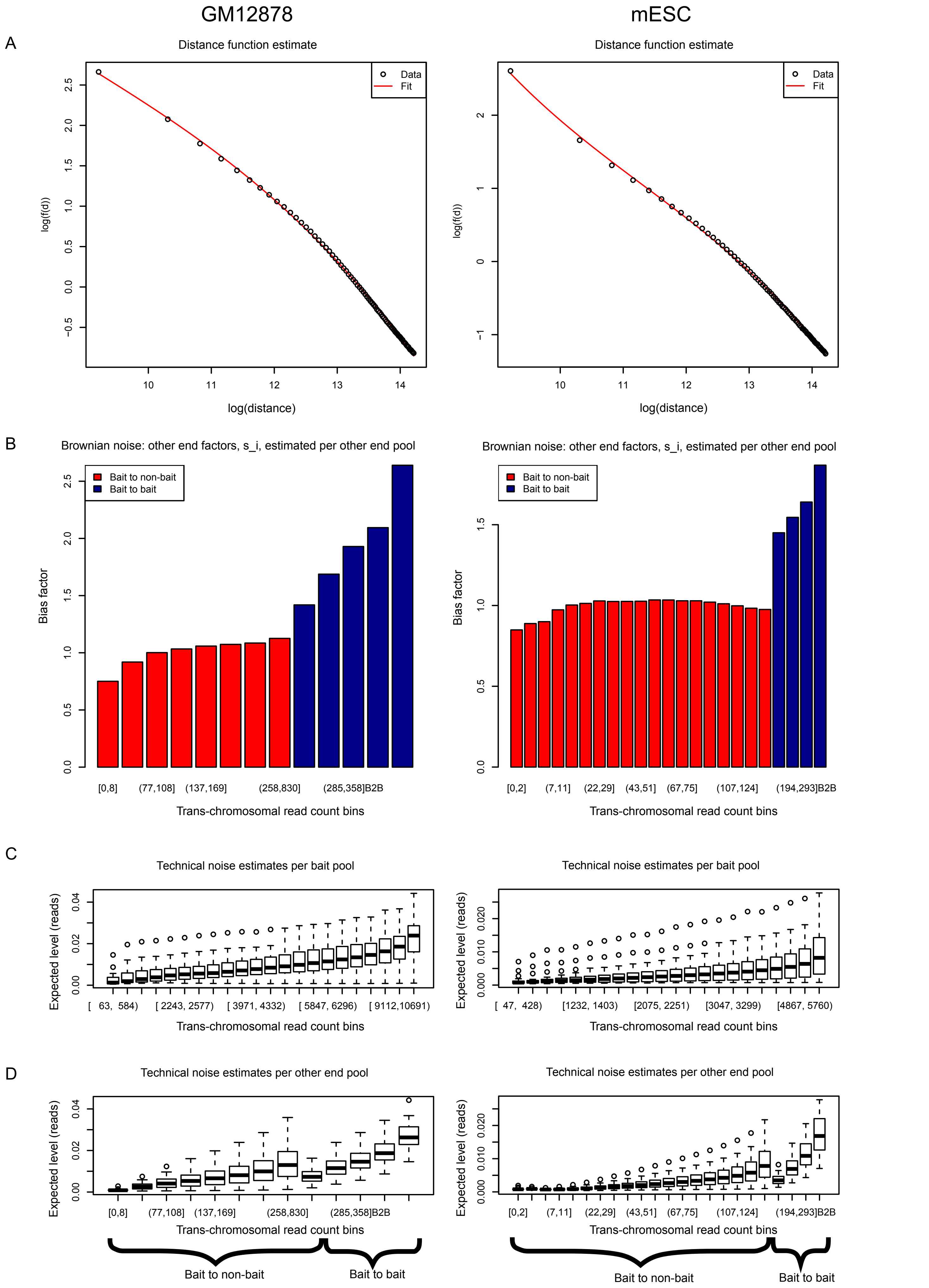

FIGURE S1
Cairns*, Freire-Pritchett*... Spivakov et al.

A

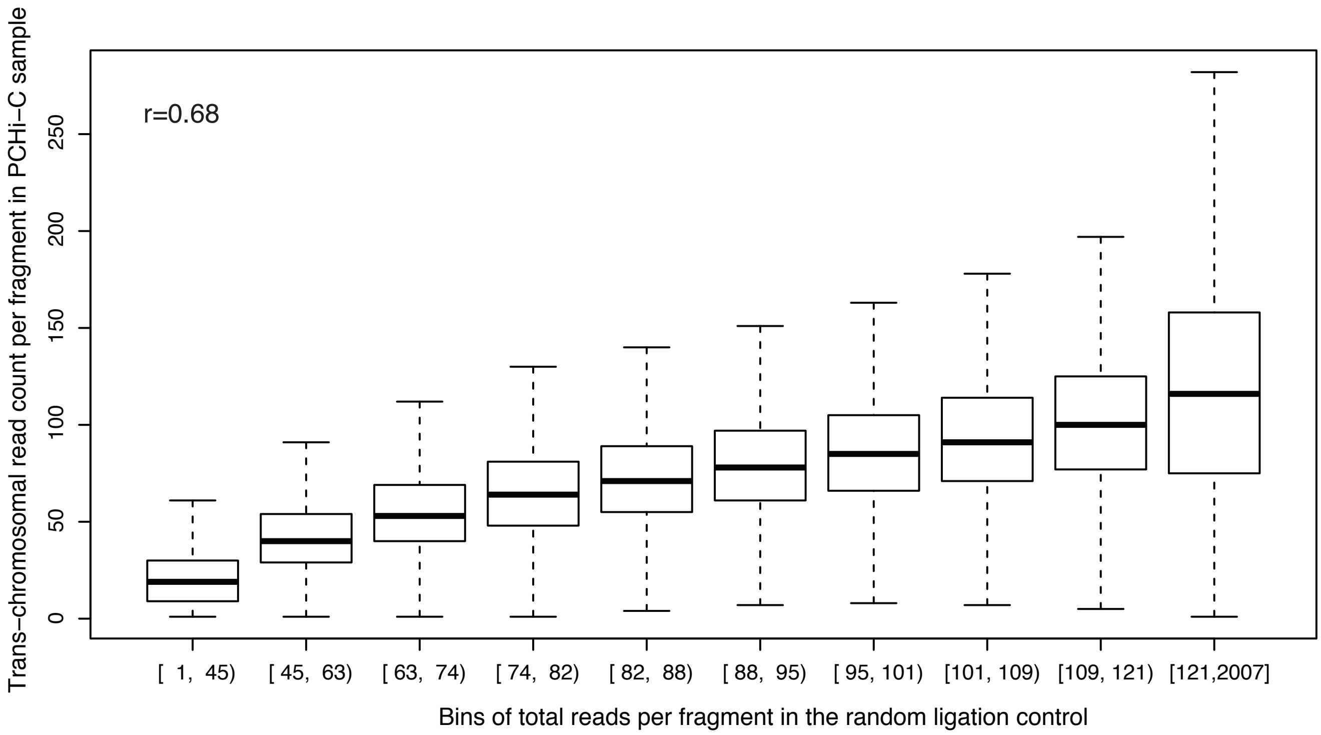

B

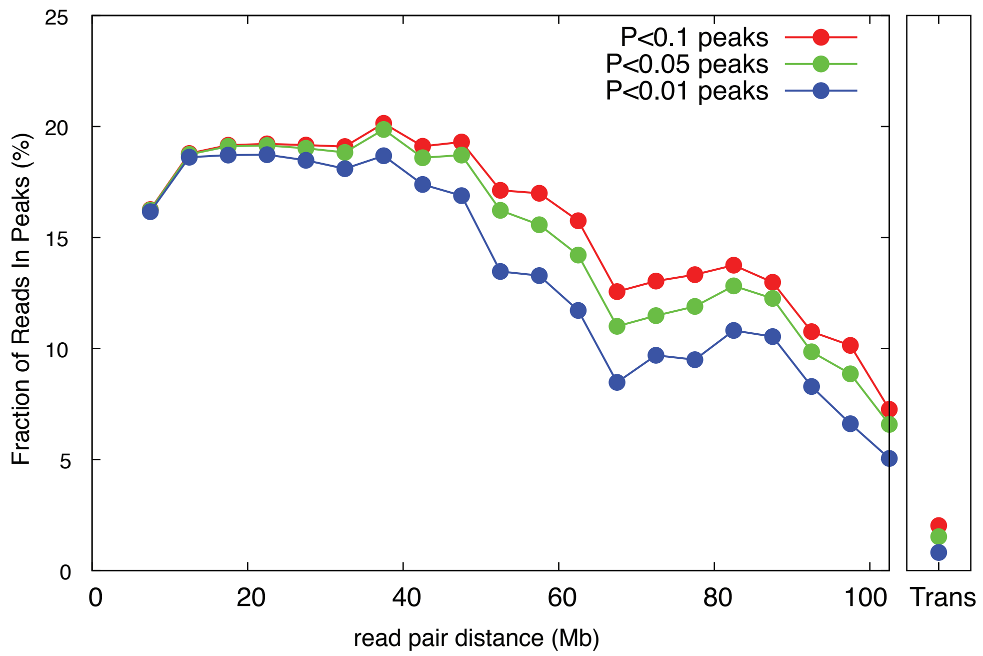

FIGURE S2
Cairns*, Freire-Pritchett*... Spivakov et al.

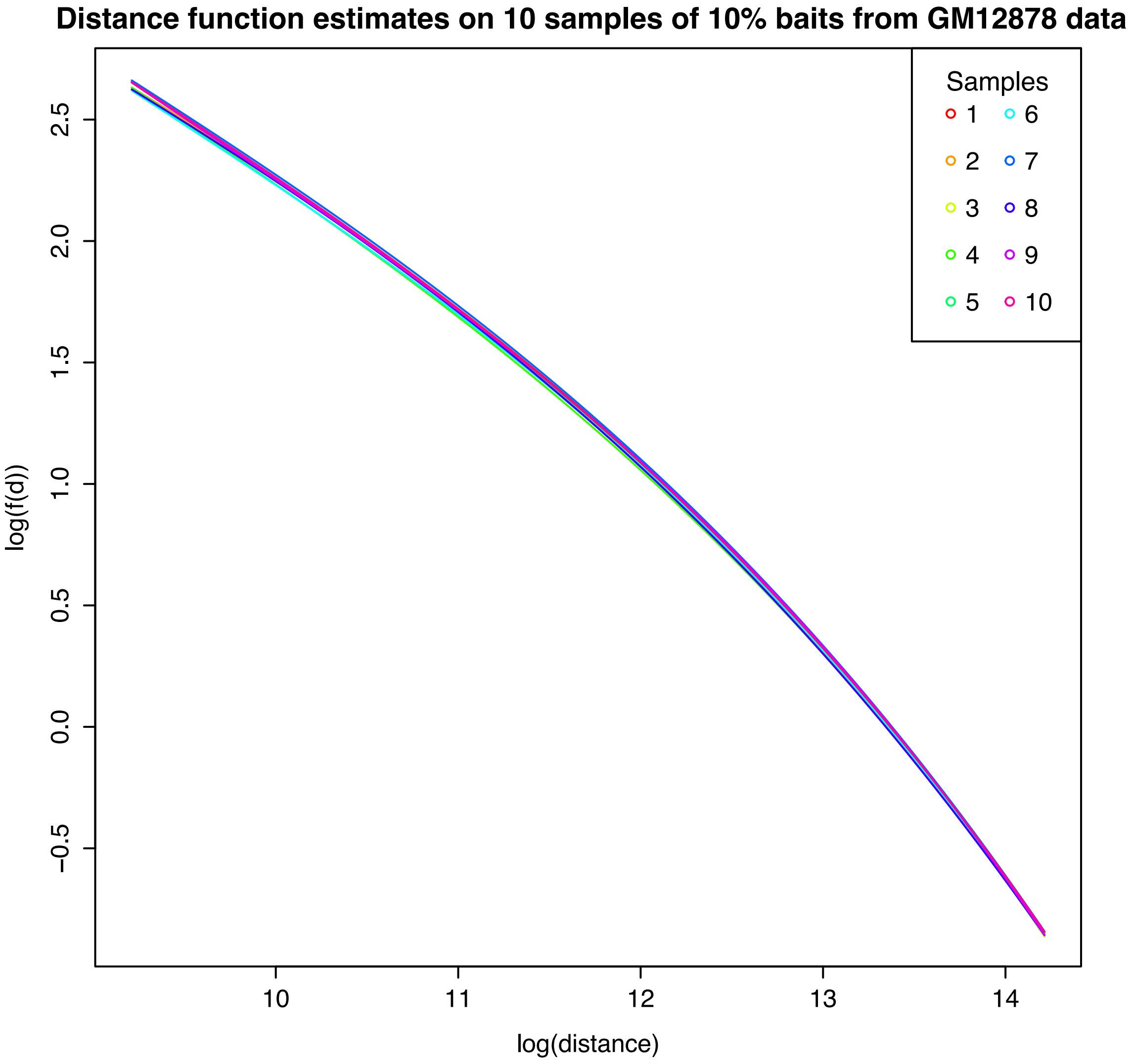

FIGURE S3
Cairns*, Freire-Pritchett*... Spivakov et al.

A

Consistency of parameter estimates on the two halves of the same GM12878 sample

NB dispersion parameter, r	3.588 / 3.617
Bait scaling factors, s_j	r=0.987
OE scaling factors, s_i	r=0.962
Expected Brownian counts, Bmean	r=0.912
Expected technical counts, Tmean	r=0.931

B

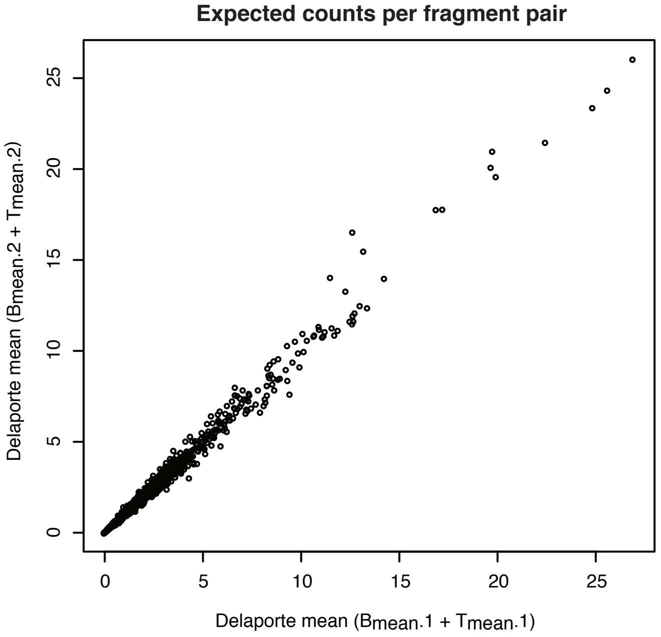

C

Two halves of the same GM12878 sample: effects of undersampling

Mean N counts	≤5	(5;10]	(10;20]	(20;30]	(30;40]	(40;50]	50+
Correlation (r) between scores	0.2	0.66	0.67	0.8	0.88	0.88	0.88
Recall of interactions (score>5), %	2	13	34	49	53	59	61

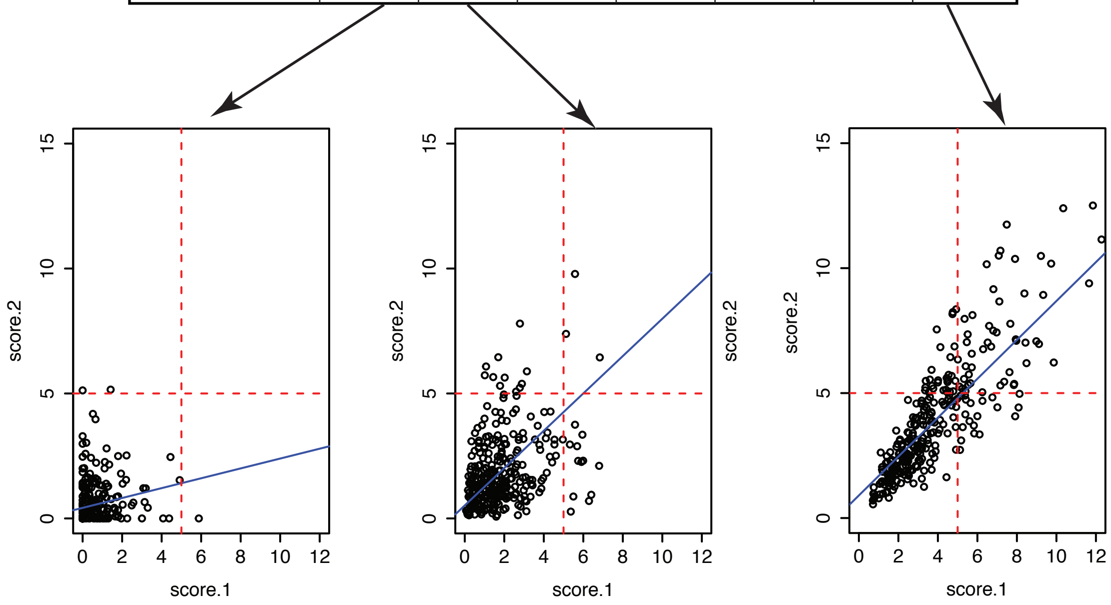

FIGURE S4
Cairns*, Freire-Pritchett*... Spivakov et al.

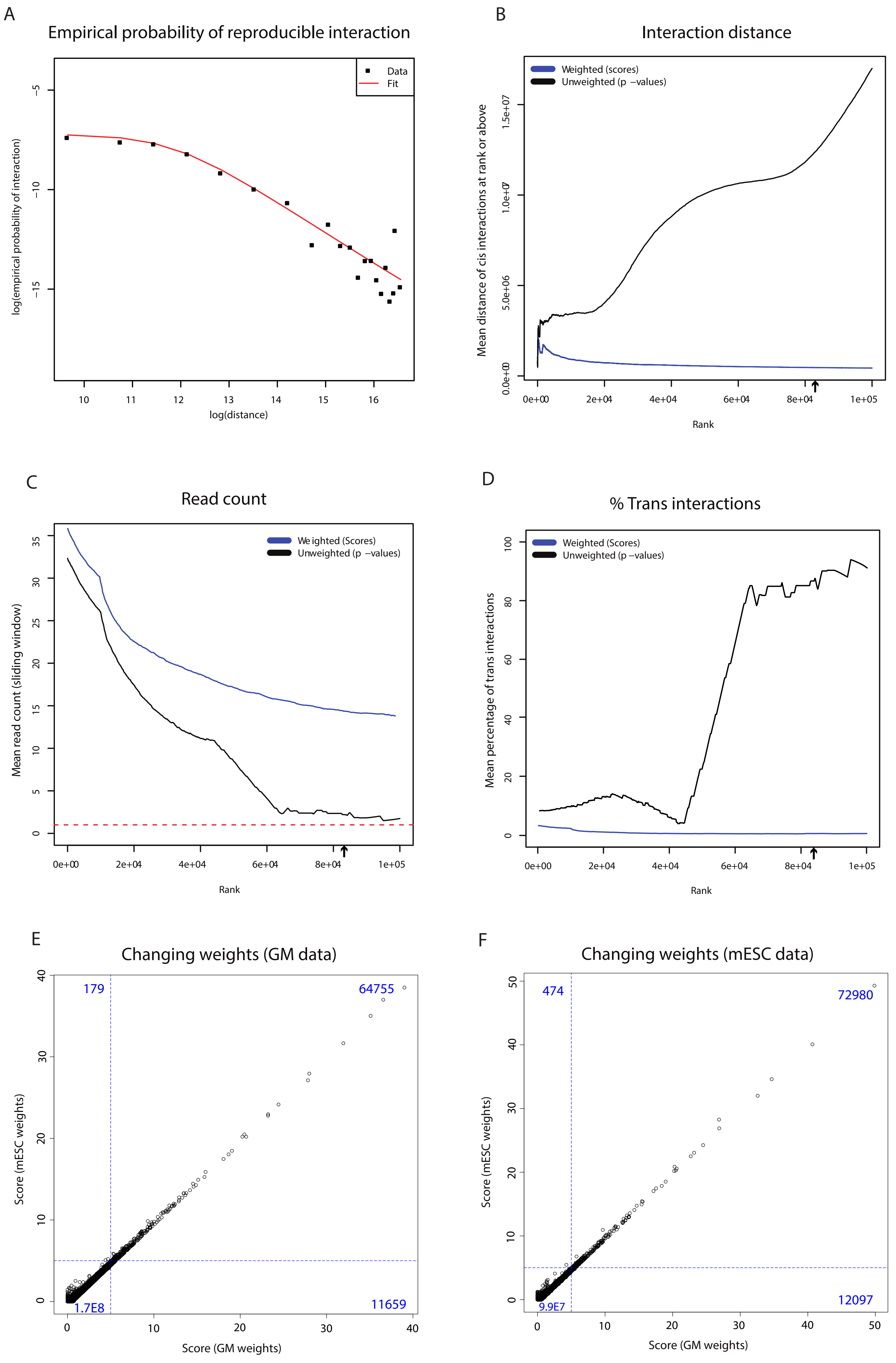

FIGURE S5
Cairns*, Freire-Pritchett*... Spivakov et al.

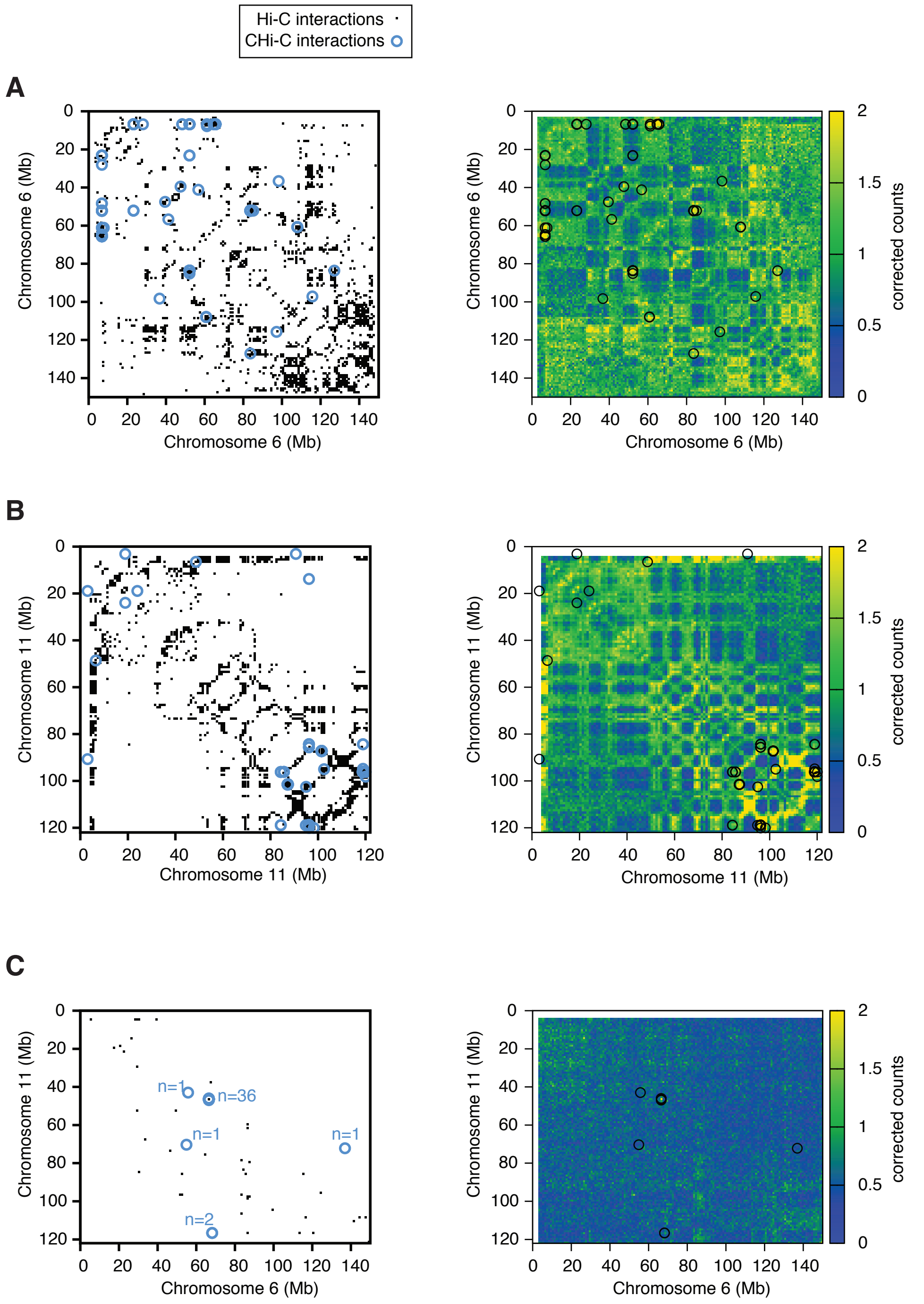

FIGURE S6
Cairns*, Freire-Pritchett*... Spivakov et al.

SUPPLEMENTARY FIGURE LEGENDS

Figure S1. Comparison of the bias factors and distance function for GM12878 (left) and mESC data (right). (A) The distance function for both cell types, plotted on a log-log scale (B) multiplicative other-end bias (each bar represents a pool of other ends defined by the numbers of trans-chromosomal read pairs accumulated by each other end; bait-to-bait interactions are pooled separately). (C-D) Technical noise is estimated separately for each combination of bait and other-end pools, each of which is defined by the number of accumulated trans-chromosomal read pairs. Here, we plot all technical noise factors for each bait (C) and other-end (D) pool, showing the distribution of technical noise levels observed for its interactions with all respective other-end or bait pools. (Data in panels A-C for GM12878 cells duplicate Fig. 4B-D and are shown here for comparison).

Figure S2. Evidence that trans-chromosomal read counts are dominated by noise. (A) Correlation between the trans-chromosomal counts accumulated by each fragment in the merged mESC CHi-C sample and the respective total per-fragment counts in the two random ligation control samples from [4] (random ligation samples were combined by pooling; boxplot outliers were not plotted). (B) Fraction of Reads in Peaks (significant interactions) detected by HOMER in the pre-capture mESC Hi-C sample from [4] at different significance thresholds. It can be seen that the overwhelming majority of trans-chromosomal read pairs map outside of detected interactions (considerably more than cis-chromosomal read pairs), suggesting they are mainly driven by noise.

Figure S3. Confirmation of the robustness of distance function estimate through cross-validation. Each line represents an f(d) estimate, on 10 data subsets, each of which consisted of 10% of the baits in the GM12878 data.

Figure S4. CHiCAGO parameter estimates are robust in the presence of undersampling. Aligned read pairs from a single replicate of GM12878 CHi-C data were randomly split into two subsamples, and parameter estimates and interaction calls were compared across these subsamples. (A-B) A table and a scatterplot showing that both parameter estimates and the resulting expected counts (Delaporte means) are highly consistent across the subsamples. (C) A table and scatterplots comparing the subsamples in terms of both the CHiCAGO scores and the thresholded interaction calls, with fragment pairs stratified by their mean read count

across the subsamples. We see that for fragment pairs with small read counts, consistency in CHiCAGO output between the subsamples is limited due to sampling error, despite consistent parameter estimates. This effect is particularly pronounced at the level of thresholded interaction calls. See Discussion for advice on handling undersampling in PCHi-C data. (r - Pearson correlation; scatterplots show random samples of 300 observations).

Figure S5. P-value weighting in mESC CHi-C data.
(A) Empirical probability of reproducible interaction (used to generate weight profiles) as a function of interaction distance generated on two replicates of mouse ES cells. (B-D) The effects of applying p-value weighting to the mESC data. The arrow on the x-axis indicates the number of significant interactions called in the weighted data. Upon applying weighting, amongst *cis*-interactions, we see a decrease in the interaction distance. Amongst all interactions, p-value weighting decreases the prevalence of trans interactions, and increases the mean read count of called interactions. Strikingly, we see that the unweighted results contain a set of high-ranking interactions that only have 1 read each. These are unlikely to be true results, and dramatically decrease in significance upon p-value weighting. (E-F) Effect of change of weights on the CHiCAGO scores. The weight profiles estimated on GM12878 or mESC data were either used for p-value weighting in their respective datasets, or swapped around. Random samples of 10,000 fragment pairs are plotted in each case; the blue dotted lines represent the default score threshold of 5. The blue numbers show how many fragment pairs (in the full dataset) pass or do not pass the threshold with each weight profile. While the swapping of weights largely retains the ranking of signals, it does have an effect on the exact identity of the interaction calls in the thresholded setting.

Figure S6. Hi-C interaction matrices for examples in Figure 10. Left: Examples of signals detected by HOMER in the Hi-C and by CHiCAGO in CHi-C for chromosomes 6, 11 and between these chromosomes in mESCs. Right: Hi-C interaction matrices showing corrected and distance-normalised read counts for the corresponding chromosomes. (Left panels duplicate Figure 10D and are shown here for comparison).

Table S1. Free parameters used in the Chicago package.

Parameter	Meaning	Default value	Rationale
adjBait2bait	Should baited fragments be treated separately?	TRUE	Baited fragments are treated separately from the rest in estimating other end-level scaling factors (s_i) and technical noise levels. It is a free parameter mainly for development purposes, and we do not recommend changing it.
binsize	The bin size (in bases) used when estimating the Brownian collision parameters.	20000	The bin size should, on average, include several (~4-5) restriction fragments to increase the robustness of parameter estimation. However, using too large bins will reduce the precision of distance function estimation. Therefore, this value needs to be changed if using an enzyme with a different cutting frequency (such as a 4-cutter).
brownianNoise.samples	Number of times subsampling occurs when estimating the Brownian collision dispersion.	5	Dispersion estimation from a subset of baits has an error attached. Averaging over multiple subsamples allows us to decrease this error. Increasing this number improves the precision of dispersion estimation at the expense of greater runtime.
brownianNoise.subset	Number of baits sampled from when estimating the Brownian collision dispersion. If set to NA, then all baits are used.	1000	Estimating dispersion from the entire dataset usually requires a prohibitively large amount of memory. A subset is chosen that is large enough to get a reasonably precise estimate of the dispersion, but small enough to stay in memory. A user with excess memory

			may wish to increase this number to further improve the estimate's precision.
maxLBrownEst	The distance range to be used for estimating the Brownian component of the null model.	1.5e6	The parameter setting should approximately reflect the maximum distance, at which the power-law distance dependence is still observable.
minFragLen / maxFragLen	These values correspond to the limits within which we observed no clear dependence between fragment length and the numbers of reads mapping to these fragments in *HindIII* CHi-C data.	150 / 40000	These parameters need to be modified when using a restriction enzyme with a different cutting frequency (such as a 4-cutter) and can also be verified by users with their datasets in each individual case. However, we note that the fragment-level scaling factors (s_i and s_j) generally incorporate the effects of fragment size, so this filtering step only aims to remove the strongest bias.
minNPerBait	Minimum number of reads that a bait has to accumulate to be included in the analysis.	250	Reasonable numbers of per-bait reads are required for robust parameter estimation. If this value is too low, the confidence of interaction calling is reduced. If too high, too many baits may be unreasonably excluded from the analysis. If it is desirable to include baits below this threshold, we recommend decreasing this parameter and then visually examining the result bait profiles (for example, using plotBaits()).
removeAdjacent	Should fragments adjacent to baits be removed from analysis?	TRUE	We remove fragments adjacent to baits by default, as the corresponding ligation products are indistinguishable from incomplete digestion. This

			setting however may be set to FALSE if the rmap and baitmap files represent bins over multiple fragments as opposed to fragment-level data (e.g., to address sparsity issues with low-coverage experiments).
tlb.filterTopPercent	Top percent of fragments with respect to accumulated trans-counts to be filtered out in the binning procedure.	0.01	Other ends are pooled together when calculating their scaling factors and as part of technical noise estimation. Binning is performed by quantile, and for the most extreme outliers this approach is not going to be adequate. Increasing this value may potentially make the estimation for the highest-count bin more robust, but will exclude additional other ends from the analysis.
tlb.minProxOEPerBin	Minimum pool size (i.e. minimum number of other ends per pool), used when pooling other ends together based on trans-counts.	1000	If this parameter is set too small, then estimates will be imprecise due to sparsity issues. If this parameter is set too large, then the model becomes inflexible and so the model fit is hindered. This parameter could be decreased in a dataset that has been sequenced to an extremely high depth. Alternatively, it may need to be decreased out of necessity, in a dataset with very few other ends - for example, the vignette decreases this setting to process the PCHiCdata package data (since these data sets span only a small subset of the genome, in each case).
tlb.minProxB2BPerBin	Minimum pool size, used when pooling	100	As per tlb.minProxOEPerBin.

	other ends together (bait-to-bait interactions only).		
techNoise.minBaitsPerBin	Minimum pool size, used when pooling baits together based on accumulated trans-counts.	1000	As per tlb.minProxOEPerBin.
