# Supplementary material for: CHiCAGO: robust detection of DNA looping interactions in Capture Hi-C data
Source: Genome Biol. 2016 Jun 15;17:127. doi: 10.1186/s13059-016-0992-2 (PMC4908757; doi:10.1186/s13059-016-0992-2)
Supplement: Additional file 3: — The CHiCAGO R package tutorial. (PDF 946 kb) [file 13059_2016_992_MOESM3_ESM.pdf]

Additional File 3: The CHiCAGO R package vignette

Jonathan Cairns*, Paula Freire-Pritchett*, Steven W. Wingett, Csilla Várnai, Andrew Dimond, Vincent Plagnol, Daniel Zerbino, Stefan Schoenfelder, Biola-Maria Javierre, Cameron Osborne, Peter Fraser and Mikhail Spivakov

****Joint lead authors***

9th May, 2016

- Introduction
- Input files required
- Example workflow
- Output plots
    - Interpreting the plots
- Output files
    - ibed format (ends with …ibed):
    - seqmonk format (ends with …seqmonk.txt):
    - washU_text format (ends with …washU_text.txt):
- Visualising interactions
- Peak enrichment for features
- Further downstream analysis
- The chicagoData object
- Using different weights
- Session info
- References

Introduction

CHiCAGO is a method for detecting statistically significant interaction events in Capture HiC data. This vignette will walk you through a typical CHiCAGO analysis.

A typical Chicago job for two biological replicates of CHi-C data takes 2-3 h wall-clock time (including sample pre-processing from bam files) and uses 50G RAM.

NOTE	A wrapper to perform this type of analysis, called *runChicago.R*, is provided as part of *chicagoTools*, which is available from our Bitbucket repository: https://bitbucket.org/chicagoTeam/chicago/src. Refer to the *chicagoTools* README for more information.

CHiCAGO uses a convolution background model accounting for both ‘Brownian collisions’ between fragments (distance-dependent) and ‘technical noise’ (distance-independent). It borrows information across interactions (with appropriate normalisation) to estimate these background components separately on different subsets of data. CHiCAGO then uses a p-value weighting procedure based on the expected true positive rates at different distance ranges (estimated from data), with scores representing soft-thresholded -log weighted p-values. The score threshold of 5 is a suggested stringent score threshold for calling significant interactions.

WARNING	The data set used in this tutorial comes from the package *PCHiCdata*. This package contains small parts (two chromosomes each) of published Promoter Capture HiC data sets in mouse ESCs (Schoenfelder et al. 2015) and GM12878 cells, derived from human LCLs (Mifsud et al. 2015) - note that both papers used a different interaction-calling algorithm and we are only reusing raw data from them. Do not use any of these sample input data for purposes other than training.

In this vignette, we use the GM12878 data (Mifsud et al. 2015). If you do not have the Chicago and PCHiCdata packages installed, you can obtain them from Bioconductor as follows:

```
source("http://www.bioconductor.org/biocLite.R")
biocLite("Chicago")
biocLite("PCHiCdata")
```

We then load the packages:

```
library(Chicago)
library(PCHiCdata)
```

Once the Chicago package is loaded, the latest version of this vignette can be obtained from within R:

```
vignette("Chicago")
```

Input files required

Before you start, you will need:

1. Five restriction map information files ("design files"):

- Restriction map file (.rmap) - a bed file containing coordinates of the restriction fragments. By default, 4 columns: chr, start, end, fragmentID.
- Bait map file (.baitmap) - a bed file containing coordinates of the baited restriction fragments, and their associated annotations. By default, 5 columns: chr, start, end, fragmentID, baitAnnotation. The regions specified in this file, including their fragmentIDs, must be an exact subset of those in the .rmap file. The baitAnnotation is a text field that is used only to annotate the output and plots.
- *nperbin* file (.npb), *nbaitsperbin* file (.nbpb), *proxOE* file (.poe) - Precompute these tables from the .rmap and .baitmap files, using the Python script `makeDesignFiles.py` from *chicagoTools* at our Bitbucket repository: https://bitbucket.org/chicagoTeam/chicago/src. Refer to the `chicagoTools` README file for more details.

We recommend that you put all five of these files into the same directory (that we refer to as designDir). An example of a valid design folder, for a two-chromosome sample of the GM12878 data used in this vignette, is provided in the PCHiCdata package, as follows.

```
dataPath <- system.file("extdata", package="PCHiCdata")
testDesignDir <- file.path(dataPath, "hg19TestDesign")
dir(testDesignDir)
```

```
## [1] "h19_chr20and21.baitmap" "h19_chr20and21.nbpb"
## [3] "h19_chr20and21.npb"     "h19_chr20and21.poe"
## [5] "h19_chr20and21.rmap"
```

NOTE	Though we talk about "restriction fragments" throughout, any non-overlapping regions can be defined in .rmap (with a subset of these defined as baits). For example, if one wanted to increase power at the cost of resolution, it is possible to use bins of restriction fragments either throughout, or for some selected regions. However, in the binned fragment framework, we advise setting *removeAdjacent* to *FALSE* - see *?setExperiment* for details on how to do this.

2. You will also need input data files, which should be in CHiCAGO input format, *.chinput*. You can obtain *.chinput* files from aligned Capture Hi-C BAM files by running `bam2chicago.sh`, available as part of `chicagoTools`. (To obtain BAM files from raw fastq files, use a Hi-C alignment & QC pipeline such as HiCUP (http://www.bioinformatics.babraham.ac.uk/projects/hicup/).

Example *.chinput* files are provided in the PCHiCdata package, as follows:

```
testDataPath <- file.path(dataPath, "GMchinputFiles")
dir(testDataPath)
```

```
## [1] "GM_rep1.chinput" "GM_rep2.chinput" "GM_rep3.chinput"
```

```
files <- c(
    file.path(testDataPath, "GM_rep1.chinput"),
    file.path(testDataPath, "GM_rep2.chinput"),
    file.path(testDataPath, "GM_rep3.chinput")
  )
```

OPTIONAL: The data set in this vignette requires some additional custom settings, both to ensure that the vignette compiles in a reasonable time and to deal with the artificially reduced size of the data set:

```
settingsFile <- file.path(system.file("extdata", package="PCHiCdata"),
                          "sGM12878Settings", "sGM12878.settingsFile")
```

Omit this step unless you know which settings you wish to alter. If you are using non-human samples, or a very unusual cell type, one set of options that you might want to change is the weighting parameters: see Using different weights.

Example workflow

We run CHiCAGO on the test data as follows. First, we create a blank `chicagoData` object, and we tell it where the design files are. For this example, we also provide the optional settings file - in your analysis, you can omit the `settingsFile` argument.

```
library(Chicago)

cd <- setExperiment(designDir = testDesignDir, settingsFile = settingsFile)
```

The properties of `chicagoData` objects are discussed more in The chicagoData object.

Next, we read in the input data files:

```
cd <- readAndMerge(files=files, cd=cd)
```

Finally, we run the pipeline with `chicagoPipeline()`:

```
cd <- chicagoPipeline(cd)
```

Output plots

`chicagoPipeline()` produces a number of plots. You can save these to disk by setting the `outprefix` argument in `chicagoPipeline()`.

The plots are as follows (an explanation follows):

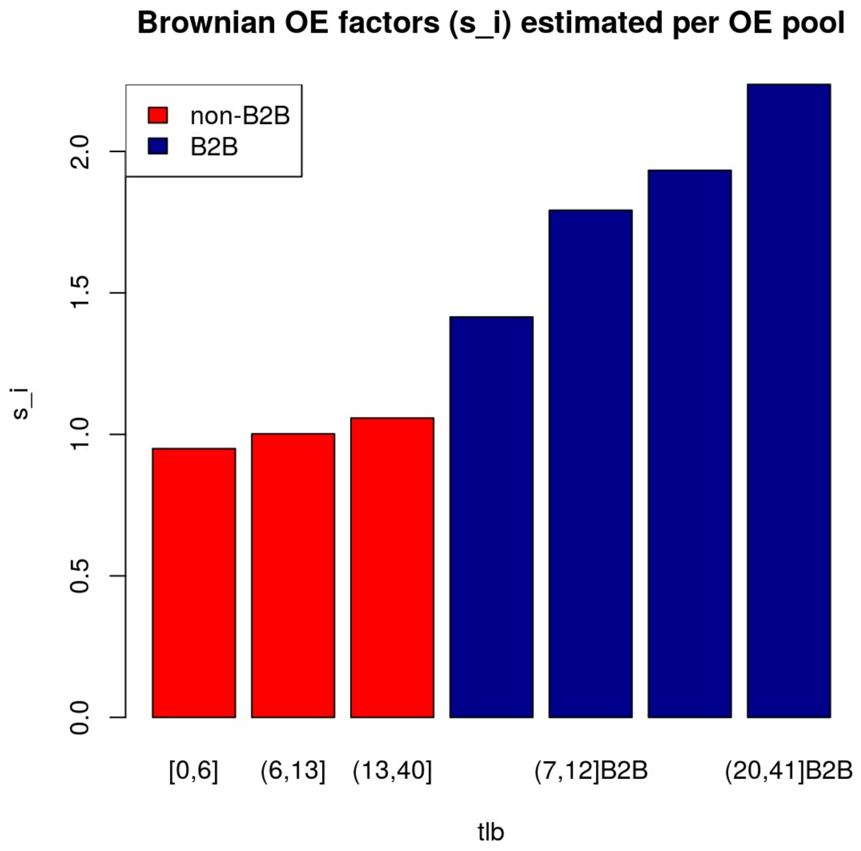

Brownian OE factors (s_i) estimated per OE pool
non-B2B
B2B
s_i
0.0
0.5
1.0
1.5
2.0
[0,6]
(6,13]
(13,40]
(7,12]B2B
(20,41]B2B
tlb

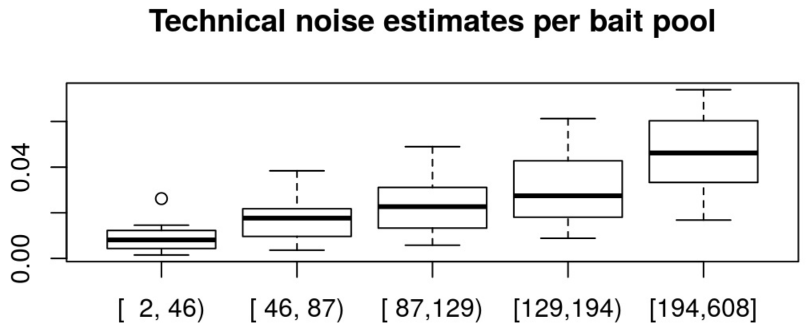

Technical noise estimates per bait pool
0.00
0.04
[2, 46)
[46, 87)
[87,129)
[129,194)
[194,608]

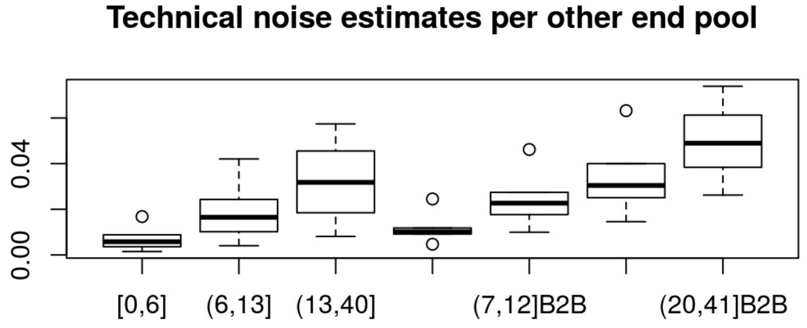

Technical noise estimates per other end pool
0.00
0.04
[0,6]
(6,13]
(13,40]
(7,12]B2B
(20,41]B2B

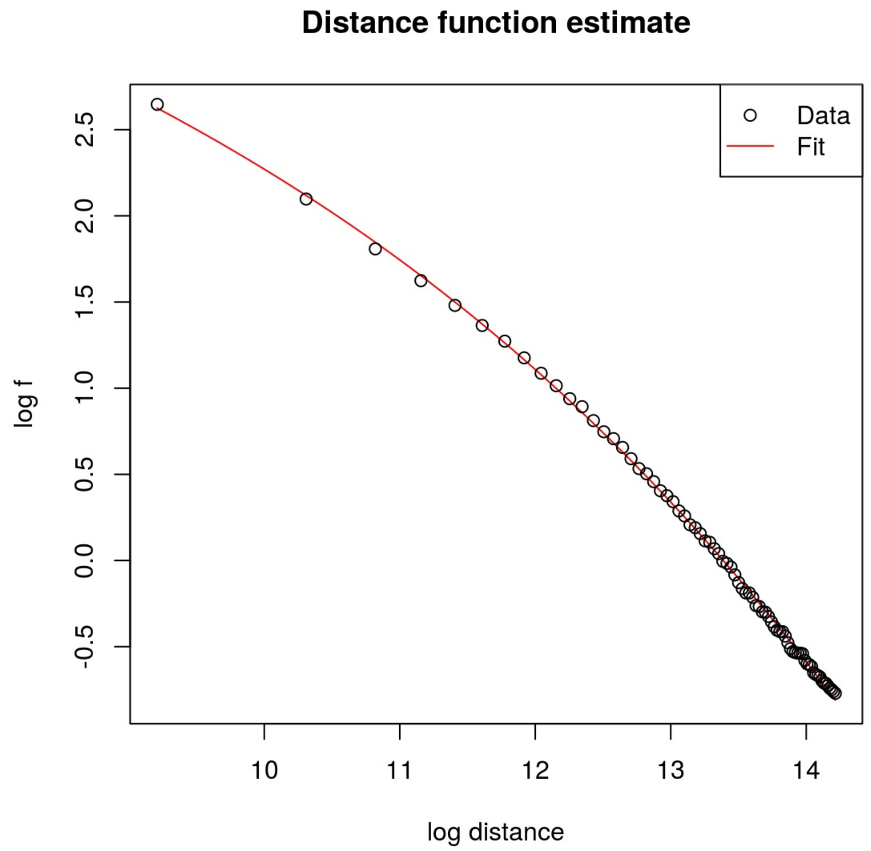

Interpreting the plots

Here, we describe the expected properties of the diagnostic plots.

Note that the diagnostic plots above are computed on the fly using only a small fraction of the full GM12878 dataset. In real-world, genome-wide datasets, more fragment pools will be visible and thus the trends described below should be more pronounced.

1. *Brownian other end factors*: The adjustment made to the mean Brownian read count, estimated in the pools of other ends. ("tlb" refers to the number of trans-chromosomal reads that the other end accumulates in total. "B2B" stands for a "bait-to-bait" interactions).

- The red bars should generally increase in height from left to right.
- The blue bars should be higher than the red bars on average, and should also increase in height from left to right.

2. *Technical noise estimates*: The mean number of technical noise reads expected for other ends and baits, respectively, per pools of fragments. These pools, displayed on the x axis, again refer to the number of trans-chromosomal reads that the fragments accumulate.

- The distributions' median and variance should trend upwards as we move from left to right.
- In the lower subplot, the bait-to-bait estimates (here, the four bars on the far right) should be higher, on average, than the others. Both groups should also have medians and variances that trend upwards, moving from left to right.

3. *Distance function*: The mean number of Brownian reads expected for an `average` bait, as a function of distance, plotted on a log-log scale.

- The function should monotonically decrease.
- The curve should fit the points reasonably well.

Output files

Two main output methods are provided. Here, we discuss the first: exporting to disk. However, it is also possible to export to a GenomeInteractions object, as described in Further downstream analysis.

You can export the results to disk, using `exportResults()`. (If you use *runChicago.R*, the files appear in ./<results-folder>/data). By default, the function outputs three different output file formats:

```
outputDirectory <- tempdir()
exportResults(cd, file.path(outputDirectory, "vignetteOutput"))
```

```
## Reading the restriction map file...
```

```
## Reading the bait map file...
```

```
## Preparing the output table...
```

```
## Writing out for seqMonk...
```

```
## Writing out interBed...
```

```
## Preprocessing for WashU outputs...
```

```
## Writing out text file for WashU browser upload...
```

Each called interaction is assigned a score that represents how strong CHiCAGO believes the interaction is (formally, it is based on -log(adjusted P-value)). Thus, a larger score represents a stronger interaction. In each case, the score threshold of 5 is applied.

Summary of output files:

ibed format (ends with ...ibed):

```
##   bait_chr bait_start bait_end                  bait_name otherEnd_chr
## 1       20     119103   138049                    DEFB126           20
## 2       20     161620   170741                    DEFB128           20
## 3       20     233983   239479                    DEFB132           20
## 4       20     268639   284501 AL034548.1;C20orf96;ZCCHC3           20
## 5       20     268639   284501 AL034548.1;C20orf96;ZCCHC3           20
## 6       20     305699   309123         RP5-1103G7.4;SOX12           20
##   otherEnd_start otherEnd_end otherEnd_name N_reads score
## 1         523682       536237       CSNK2A1       6  6.01
## 2          73978        76092             .      16  5.18
## 3         206075       209203       DEFB129      33  5.37
## 4         293143       304037             .      34  7.29
## 5         304038       305698             .      34  8.81
## 6         293143       304037             .      37  5.51
```

- each row represents an interaction
- first four columns give information about the chromosome, start, end and name of the bait fragment
- next four columns give information about the chromosome, start, end and name of the other end that interacts with the bait fragment
- N_reads is the number of reads
- score is as defined above

seqmonk format (ends with ...seqmonk.txt):

```
##   V1     V2     V3      V4 V5   V6
## 1 20 119103 138049 DEFB126  6 6.01
## 2 20 523682 536237 CSNK2A1  6 6.01
## 3 20 161620 170741 DEFB128 16 5.18
## 4 20  73978  76092       . 16 5.18
## 5 20 233983 239479 DEFB132 33 5.37
## 6 20 206075 209203 DEFB129 33 5.37
```

- Can be read by seqmonk (http://www.bioinformatics.babraham.ac.uk/projects/seqmonk/).
- An interaction is represented by two rows: the first row is the bait, the second the other end. Thus, the file alternates: bait1, otherEnd1, bait2, otherEnd2, …
- Columns are: chromosome, start, end, name, number of reads, interaction score (see above)

washU_text format (ends with …washU_text.txt):

```
##                   V1                  V2   V3
## 1 chr20,119103,138049 chr20,523682,536237 6.01
## 2 chr20,161620,170741   chr20,73978,76092 5.18
## 3 chr20,233983,239479 chr20,206075,209203 5.37
## 4 chr20,268639,284501 chr20,293143,304037 7.29
## 5 chr20,268639,284501 chr20,304038,305698 8.81
## 6 chr20,305699,309123 chr20,293143,304037 5.51
```

- Can be read by the WashU browser (http://epigenomegateway.wustl.edu).
- Upload via the "Got text files instead? Upload them from your computer" link.
- Note - Advanced users may wish to export to washU_track format instead. See the help page for `exportResults()`.

For bait-to-bait interactions, the interaction can be tested either way round (i.e. either fragment can be considered the "bait"). In most output formats, both of these tests are preserved. The exception is washU output, where these scores are consolidated by taking the maximum.

NOTE

When comparing interactions detected between multiple replicates, the degree of overlap may appear to be lower than expected. This is due to the undersampled nature of most CHi-C datasets. Sampling error can drive down the sensitivity, particularly for interactions that span large distances and have low read counts. As such, low overlap is not necessarily an indication of a high false discovery rate.

Undersampling needs to be taken into consideration when interpreting CHiCAGO results. In particular, we recommend performing comparisons at the score-level rather than at the level of thresholded interaction calls. Potentially, differential analysis algorithms for sequencing data such as *DESeq2* (Love, Huber, and Anders 2014) may also be used to formally compare the enrichment at CHiCAGO-detected interactions between conditions at the count level, although power will generally be a limiting factor.

Formal methods such as *sdef* (Blangiardo, Cassese, and Richardson 2010) may provide a more balanced view of the consistency between replicates. Alternatively, additional filtering based on the mean number of reads per detected interaction (e.g. removing calls with N<10 reads) will reduce the impact of undersampling on the observed overlap, but at the cost of decreasing the power to detect longer-range interactions.

Visualising interactions

The `plotBaits()` function can be used to plot the raw read counts versus linear distance from bait for either specific or random baits, labelling significant interactions in a different colour. By default, 16 random baits are plotted, with interactions within 1 Mb from bait passing the threshold of 5 shown in red and those passing the more lenient threshold of 3 shown in blue.

```
plottedBaitIDs <- plotBaits(cd, n=6)
```

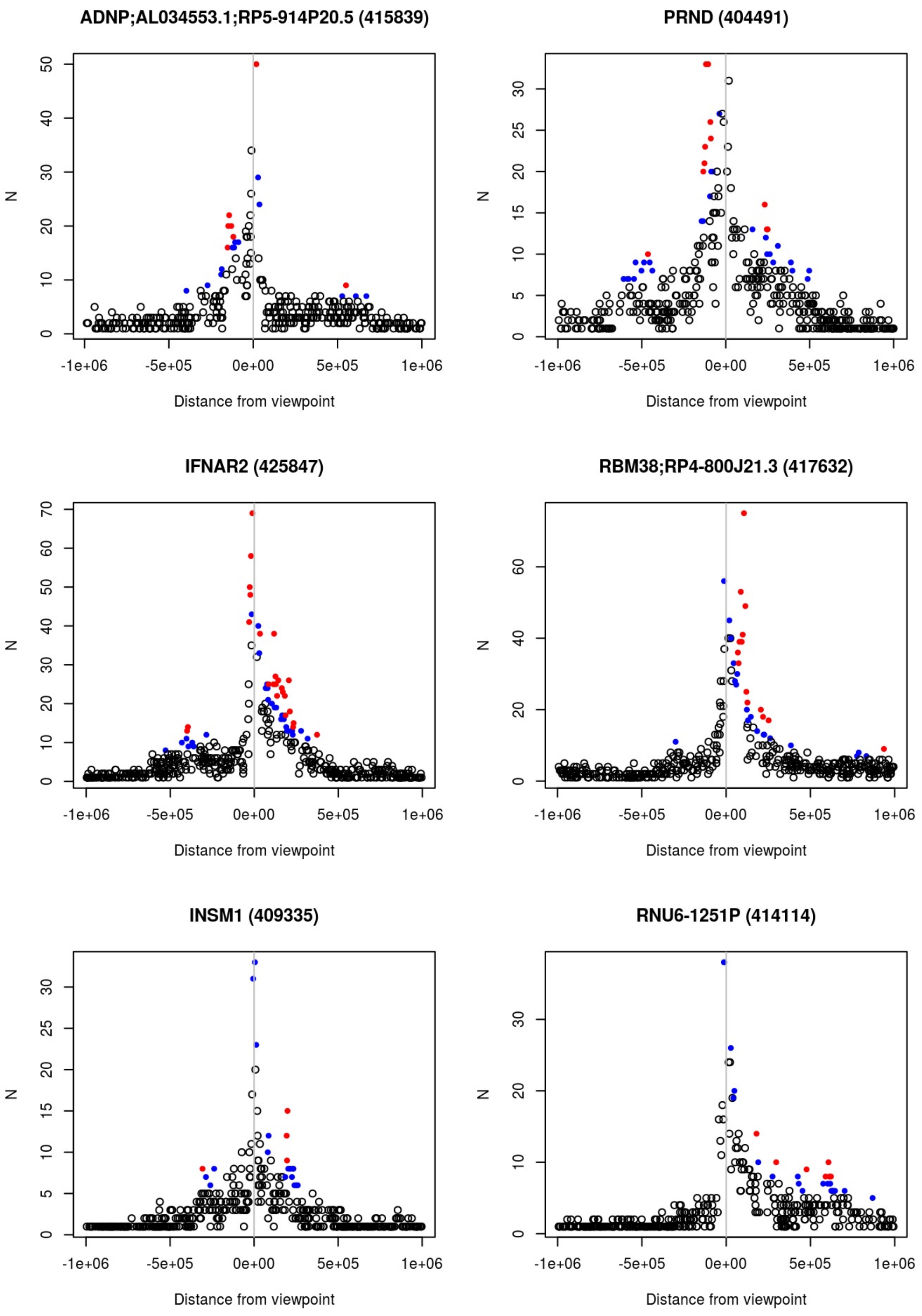

Peak enrichment for features

`peakEnrichment4Features()` tests the hypothesis that other ends in the CHiCAGO output are enriched for genomic features of interest - for example, histone marks associated with enhancers. We find out how many overlaps are expected under the null hypothesis (i.e. that there is no enrichment) by shuffling the other ends around in the genome, while preserving the overall distribution of distances over which interactions span.

You will need additional files to perform this analysis - namely, a .bed file for each feature. We include ChIP-seq data from the ENCODE consortium (The ENCODE Project Consortium 2012), also restricted to chr20 and chr21. (Data accession numbers: Bernstein lab GSM733752, GSM733772, GSM733708, GSM733664, GSM733771, GSM733758)

First, we find the folder that contains the features, and construct a list of the features to use:

```
featuresFolder <- file.path(dataPath, "GMfeatures")
dir(featuresFolder)
```

```
## [1] "featuresGM.txt"
## [2] "spp.wgEncodeBroadHistoneGm12878CtcfStdAln_chr20and21.narrowPeak"
## [3] "wgEncodeBroadHistoneGm12878H3k27acStdAln_chr20and21.narrowPeak"
## [4] "wgEncodeBroadHistoneGm12878H3k27me3StdAln_chr20and21.narrowPeak"
## [5] "wgEncodeBroadHistoneGm12878H3k4me1StdAln_chr20and21.narrowPeak"
## [6] "wgEncodeBroadHistoneGm12878H3k4me3StdAln_chr20and21.narrowPeak"
## [7] "wgEncodeBroadHistoneGm12878H3k9me3StdAln_chr20and21.narrowPeak"
```

```
featuresFile <- file.path(featuresFolder, "featuresGM.txt")
featuresTable <- read.delim(featuresFile, header=FALSE, as.is=TRUE)
featuresList <- as.list(featuresTable$V2)
names(featuresList) <- featuresTable$V1
featuresList
```

```
## $CTCF
## [1] "spp.wgEncodeBroadHistoneGm12878CtcfStdAln_chr20and21.narrowPeak"
##
## $H3K4me1
## [1] "wgEncodeBroadHistoneGm12878H3k4me1StdAln_chr20and21.narrowPeak"
##
## $H3K4me3
## [1] "wgEncodeBroadHistoneGm12878H3k4me3StdAln_chr20and21.narrowPeak"
##
## $H3k27ac
## [1] "wgEncodeBroadHistoneGm12878H3k27acStdAln_chr20and21.narrowPeak"
##
## $H3K27me3
## [1] "wgEncodeBroadHistoneGm12878H3k27me3StdAln_chr20and21.narrowPeak"
##
## $H3K9me3
## [1] "wgEncodeBroadHistoneGm12878H3k9me3StdAln_chr20and21.narrowPeak"
```

Next, we feed this information into the `peakEnrichment4Features()` function.

As part of the analysis, `peakEnrichment4Features()` takes a distance range (by default, the full distance range over which interactions are observed), and divides it into some number of bins. We must select the number of bins; here, we choose that number to ensure that the bin size is approximately 10kb. If the defaults are changed, a different number of bins is more appropriate. See `?peakEnrichment4Features` for more information.

```
no_bins <- ceiling(max(abs(intData(cd)$distSign), na.rm = TRUE)/1e4)

enrichmentResults <- peakEnrichment4Features(cd, folder=featuresFolder,
              list_frag=featuresList, no_bins=no_bins, sample_number=100)
```

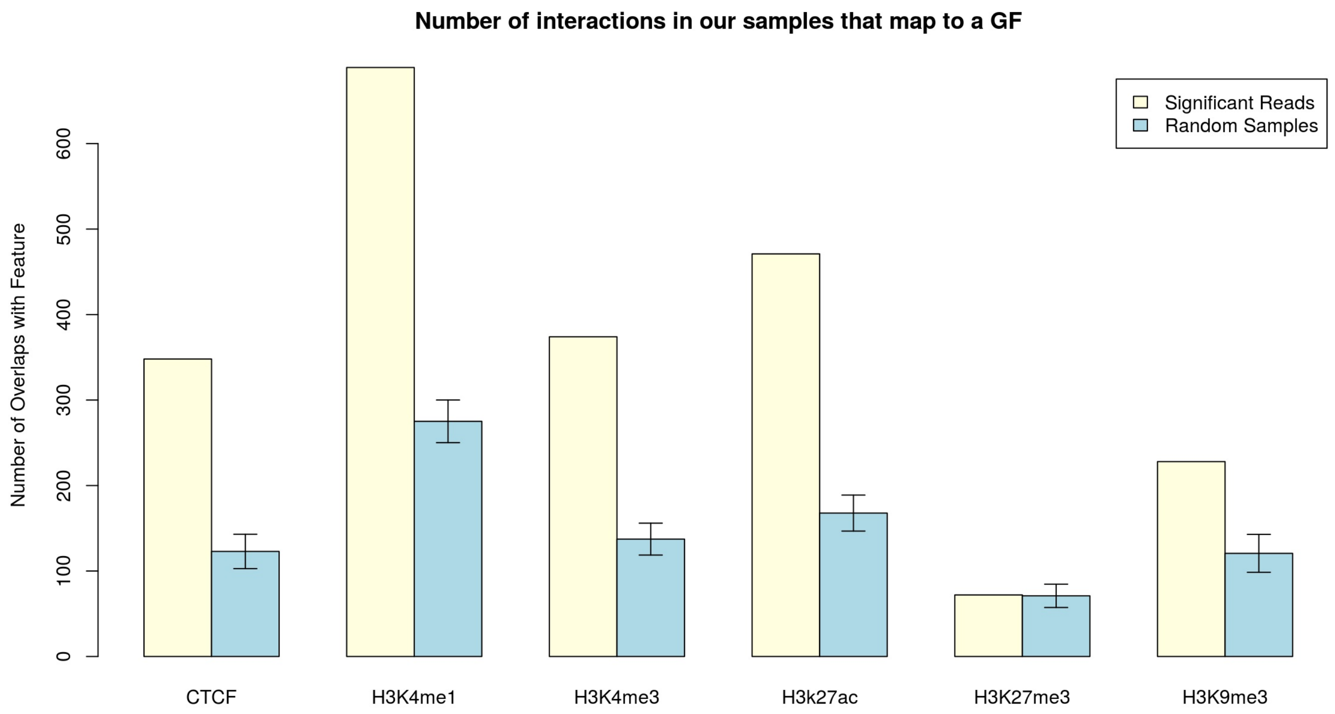

Note the plot produced by this function. For each feature type, the yellow bar represents the number of features that overlap with interaction other ends. The blue bar represents what would be expected by chance, with a 95% confidence interval for the mean number of overlaps plotted. If the yellow bar lies outside of this interval, we reject the null hypothesis, thus concluding that there is enrichment/depletion of that feature.

The information displayed in the plot is also returned in tabular form (OL = Overlap, SI = Significant Interactions, SD = Standard Deviation, CI = Confidence Interval):

```
enrichmentResults
```

```
##          OLwithSI MeanOLwithSamples SDOLwithSample   LowerCI  HigherCI
## CTCF          348            122.92      10.254026 102.82211 143.01789
## H3K4me1       689            275.10      12.729112 250.15094 300.04906
## H3K4me3       374            137.32       9.526020 118.64900 155.99100
## H3k27ac       471            167.77      10.742021 146.71564 188.82436
## H3K27me3       72             70.99       6.956568  57.35513  84.62487
## H3K9me3       228            120.66      11.316583  98.47950 142.84050
```

Further downstream analysis

We can perform further downstream analysis in R or Bioconductor, using functionality from the *GenomicInteractions* package. First, we export the significant interactions into a GenomicInteractions object:

```
library(GenomicInteractions)
```

```
## Warning: replacing previous import by 'ggplot2::Position' when loading
## 'GenomicInteractions'
```

```
library(GenomicRanges)
gi <- exportToGI(cd)
```

From here, we can pass the CHiCAGO results through to other Bioconductor functionality. In the following example, we

find out which other ends overlap with the H3K4me1 enhancer mark, using ENCODE data. We use *AnnotationHub* to fetch a relevant enhancer mark track from the ENCODE project:

```
library(AnnotationHub)
ah <- AnnotationHub()
hs <- query(ah, c("GRanges", "EncodeDCC", "Homo sapiens", "H3k4me1"))
enhancerTrack <- hs[["AH23254"]]
```

Next, we use the `anchorTwo()` function to extract the other end locations from the GenomicInteractions object (`anchorOne()` would give us the bait locations instead). Note that in this particular instance, the `seqlevels()` also need to be changed before performing the comparison, adding “chr” to make them match those of the annotation.

```
otherEnds <- anchorTwo(gi)
otherEnds <- renameSeqlevels(otherEnds, c("chr20","chr21"))
```

Finally, we look at which other ends overlap the enhancer marks:

```
findOverlaps(otherEnds, enhancerTrack)
```

```
## Hits object with 4593 hits and 0 metadata columns:
##          queryHits subjectHits
##          <integer>   <integer>
##      [1]         1       40732
##      [2]         1       40735
##      [3]         8       40717
##      [4]         8       40718
##      [5]         9       40721
##      ...       ...         ...
##   [4589]      4195       17630
##   [4590]      4195       17631
##   [4591]      4196       17676
##   [4592]      4196       17677
##   [4593]      4199       17665
##   -------
##   queryLength: 4200
##   subjectLength: 109612
```

Further note that the annotation’s genome version should match that of the promoter capture data, namely hg19:

```
hs["AH23254"]$genome
```

```
## [1] "hg19"
```

The chicagoData object

In the above workflows, *cd* is a *chicagoData* object. It contains three elements:

- `intData(cd)` is a *data.table* (note: not a *data.frame*) that contains information about fragment pairs.
- `settings(cd)` is a list of settings, usually set with the setExperiment() function.
- `params(cd)` is a list of parameters. This list is populated as the pipeline runs, and CHiCAGO estimates them in turn.

A closer look at `intData(cd)`:

```
head(intData(cd), 2)
```

```
##    baitID otherEndID distbin       s_j otherEndLen distSign isBait2bait
## 1: 403463     403833      NA 0.2368791        2579  1652804       FALSE
## 2: 403463     403843      NA 0.2368791        6302  1690808       FALSE
##    N.1 N.2 N.3 N refBinMean       s_i NNb NNboe    tlb      tblb
## 1:   0   1   0 1         NA 0.9494934   4     4  [0,6] [  2, 46)
## 2:   0   1   0 1         NA 1.0021336   4     4 (6,13] [  2, 46)
##          Tmean      Bmean     log.p    log.w     log.q score
## 1: 0.001485762 0.08970408 -2.443258 1.406451 -3.849708     0
## 2: 0.004021026 0.09243682 -2.389692 1.350609 -3.740300     0
```

Columns:

- baitID: ID of baited fragment
- otherEndID: ID of other end fragment
- s_j: bait-specific scaling factor (Brownian component)
- otherEndLen: The length of the other end fragment
- distSign: The distance from the baited fragment to the other end fragment. Positive and negative values indicate that the other end is, respectively, $5'$ and $3'$ of the baited fragment. NA indicates a trans interactions.
- isBait2Bait: TRUE if the other end fragment is also a baited fragment
- N.1, N.2, …: Raw read counts per replicate (see `?mergeSamples`).
- N: Merged count (see `?mergeSamples`) or raw count in the case of single-replicate interaction calling.
- refBinMean: Can be ignored. (see `?normaliseBaits`)
- s_i: other end-specific scaling factor (Brownian component)
- NNb: “N normalised for baits”, a count scaled up by accounting for s_j. May be useful for visualization.
- NNboe: “N normalised for baits and other ends”; may be useful for visualization.
- tlb: Class of other end, based on the number of fragments on other chromosomes that have read pairs.
- tblb: As tlb, for the bait fragment.
- Tmean: Expected count from technical noise.
- Bmean: Expected count from Brownian component. (Thus, the expected count under the null hypothesis is Tmean + Bmean.)
- log.p: p-value associated with fragment pair, on log-scale.
- log.w: p-value weight, on log-scale.
- log.q: weighted p-value, on log-scale.
- score: Final CHiCAGO score.

WARNING: Many functions in CHiCAGO update `intData(cd)` by reference, which means that `intData(cd)` can change even when you do not explicitly assign to it. To avoid this behaviour, copy the *chicagoData* object first:

```
newCd = copyCD(cd)
```

Using different weights

CHiCAGO uses a p-value weighting procedure to upweight proximal interactions and downweight distal interactions. This procedure has four tuning parameters.

The default values of these tuning parameters were calibrated on calls from seven human Macrophage data sets. Provided that your cell type is not too dissimilar to these calibration data, it should be fine to leave the parameters at their default settings. However, if your data set is from a different species or an unusual cell type, you may wish to recalibrate these parameters using data from cell types similar to yours. You can do this with the fitDistCurve.R script in *chicagoTools*, which we demonstrate in this section.

First, run all of the samples through `chicagoPipeline()`, saving each `chicagoData` object in individual .rds files (see `saveRDS()`). Alternatively, if you are using the runChicago.R wrapper, .rds files should be generated automatically.

Second, run the fitDistCurve.R script. As an example, if we had three biological replicates of mESC cells, we might run the following script at the Unix command prompt:

```
Rscript fitDistCurve.R mESC --inputs mESC1.rds,mESC2.rds,mESC3.rds
```

This script produces the file mESC.settingsFile, which you can read in to `modifySettings()` as usual - see the Input files

required section.

Additionally, the script produces a plot (in this case, called mESC_mediancurveFit.pdf) that can be used to diagnose unreliable estimates. By default, five coloured lines are shown, each representing a parameter estimate from a subset of the data. An unreliable fit is typically diagnosed when the coloured lines are highly dissimilar to each other, and thus the black median line is not representative of them. (Some dissimilarity may be OK, since the median confers robustness.)

For the user's convenience, a set of precomputed weights are also provided in the package:

```
weightsPath <- file.path(system.file("extdata", package="Chicago"),
                          "weights")
dir(weightsPath)
```

```
## [1] "GM12878-2reps.settings"          "humanMacrophage-7reps.settings"
## [3] "mESC-2reps.settings"
```

For example, to use the GM12878 weights, supply the appropriate settings file to `setExperiment()` as per the following:

```
weightSettings <- file.path(weightsPath, "GM12878-2reps.settings")
cd <- setExperiment(designDir = testDesignDir, settingsFile = weightSettings)
```

Session info

```
sessionInfo()
```

```
## R version 3.2.2 (2015-08-14)
## Platform: x86_64-pc-linux-gnu (64-bit)
## Running under: Ubuntu 14.04.4 LTS
##
## locale:
##  [1] LC_CTYPE=en_GB.UTF-8       LC_NUMERIC=C
##  [3] LC_TIME=en_GB.UTF-8        LC_COLLATE=en_GB.UTF-8
##  [5] LC_MONETARY=en_GB.UTF-8    LC_MESSAGES=en_GB.UTF-8
##  [7] LC_PAPER=en_GB.UTF-8       LC_NAME=C
##  [9] LC_ADDRESS=C               LC_TELEPHONE=C
## [11] LC_MEASUREMENT=en_GB.UTF-8 LC_IDENTIFICATION=C
##
## attached base packages:
## [1] stats4    parallel  stats     graphics  grDevices utils     datasets
## [8] methods   base
##
## other attached packages:
##  [1] rtracklayer_1.28.10       AnnotationHub_2.0.4
##  [3] GenomicRanges_1.20.8      GenomeInfoDb_1.4.3
##  [5] IRanges_2.2.9             S4Vectors_0.6.6
##  [7] BiocGenerics_0.14.0       GenomicInteractions_1.2.3
##  [9] PCHiCdata_1.0.0           Chicago_1.0.1
## [11] data.table_1.9.6          BiocStyle_1.6.0
##
## loaded via a namespace (and not attached):
##  [1] Rcpp_0.12.5                  lattice_0.20-33
##  [3] Delaporte_2.2-3              Rsamtools_1.20.5
##  [5] Biostrings_2.36.4            assertthat_0.1
##  [7] digest_0.6.9                 mime_0.4
##  [9] R6_2.1.2                     plyr_1.8.3
## [11] chron_2.3-47                 futile.options_1.0.0
## [13] acepack_1.3-3.3              RSQLite_1.0.0
## [15] evaluate_0.9                 BiocInstaller_1.18.5
## [17] httr_1.1.0                   ggplot2_2.1.0
## [19] zlibbioc_1.14.0              curl_0.9.7
## [21] rpart_4.1-10                 Matrix_1.2-6
## [23] rmarkdown_0.9.6              splines_3.2.2
## [25] BiocParallel_1.2.22          stringr_1.0.0
## [27] foreign_0.8-66               igraph_1.0.1
## [29] RCurl_1.95-4.8               munsell_0.4.3
## [31] shiny_0.13.2                 httpuv_1.3.3
## [33] htmltools_0.3.5              nnet_7.3-12
## [35] interactiveDisplayBase_1.6.1 gridExtra_2.2.1
## [37] Hmisc_3.17-4                 matrixStats_0.50.2
## [39] XML_3.98-1.4                 dplyr_0.4.3
## [41] GenomicAlignments_1.4.2      MASS_7.3-45
## [43] bitops_1.0-6                 grid_3.2.2
## [45] xtable_1.8-2                 gtable_0.2.0
## [47] DBI_0.4-1                    magrittr_1.5
## [49] formatR_1.4                  scales_0.4.0
## [51] stringi_1.0-1                XVector_0.8.0
## [53] latticeExtra_0.6-28          futile.logger_1.4.1
## [55] Formula_1.2-1                lambda.r_1.1.7
## [57] RColorBrewer_1.1-2           tools_3.2.2
## [59] Biobase_2.28.0               plotrix_3.6-2
## [61] survival_2.39-4              yaml_2.1.13
## [63] AnnotationDbi_1.30.1         colorspace_1.2-6
## [65] cluster_2.0.4                knitr_1.13
```

References

Blangiardo, Marta, Alberto Cassese, and Sylvia Richardson. 2010. "sdef: An R Package to Synthesize Lists of Significant

Features in Related Experiments." *BMC Bioinformatics* 11 (1). BioMed Central Ltd: 270.

Love, Michael I, Wolfgang Huber, and Simon Anders. 2014. "Moderated Estimation of Fold Change and Dispersion for RNA-Seq Data with DESeq2." *Genome Biology* 15 (12): 550. doi:10.1186/s13059-014-0550-8.

Mifsud, Borbala, Filipe Tavares-Cadete, Alice N Young, Robert Sugar, Stefan Schoenfelder, Lauren Ferreira, Steven W Wingett, et al. 2015. "Mapping Long-Range Promoter Contacts in Human Cells with High-Resolution Capture Hi-C." *Nature Genetics* 47 (6). Nature Publishing Group: 598–606.

Schoenfelder, Stefan, Mayra Furlan-Magaril, Borbala Mifsud, Filipe Tavares-Cadete, Robert Sugar, Biola-Maria Javierre, Takashi Nagano, et al. 2015. "The Pluripotent Regulatory Circuitry Connecting Promoters to Their Long-Range Interacting Elements." *Genome Research* 25 (4). Cold Spring Harbor Lab: 582–97.

The ENCODE Project Consortium. 2012. "An Integrated Encyclopedia of DNA Elements in the Human Genome." *Nature* 489 (7414): 57–74.

Loading [MathJax]/jax/output/HTML-CSS/fonts/TeX/fontdata.js
